# Th1 Disabled Function in Response to TLR4 Stimulation of Monocyte-Derived DC from Patients Chronically-Infected by Hepatitis C Virus

**DOI:** 10.1371/journal.pone.0002260

**Published:** 2008-05-28

**Authors:** Laure Perrin-Cocon, Sophie Agaugué, Olivier Diaz, Béatrice Vanbervliet, Sandra Dollet, Aurélie Guironnet-Paquet, Patrice André, Vincent Lotteau

**Affiliations:** 1 Inserm, U851, Lyon, France; 2 Université de Lyon, IFR128 BioSciences Lyon-Gerland, Lyon, France; 3 Hospices civils de Lyon, Hôpital de la croix rousse, Laboratoire de virologie, Lyon, France; Cambridge University, United Kingdom

## Abstract

**Background:**

Lack of protective antibodies and inefficient cytotoxic responses are characteristics of chronic hepatitis C infection. A defect in dendritic cell (DC) function has thus been suspected, but this remains a controversial issue.

**Methods and Findings:**

Here we show that monocyte-derived DC (MoDC) from chronically-infected patients can mature in response to TLR1/2, TLR2/6 or TLR3 ligands. In contrast, when stimulated with the TLR4 ligand LPS, MoDC from patients show a profound defect in inducing IFNγ secretion by allogeneic T cells. This defect is not due to defective phenotypic maturation or to the presence of HCV-RNA in DC or monocytes but is correlated to reduced IL-12 secretion by DC. Restoration of DC ability to stimulate IFNγ secretion can be obtained by blocking MEK activation in DC, indicating that MEK/ERK pathway is involved in the Th1 defect of MoDC. Monocytes from HCV patients present increased spontaneous secretion of cytokines and chemokines, especially MIP-1β. Addition of MIP-1β on healthy monocytes during differentiation results in DC that have Th1 defect characteristic of MoDC from HCV patients, suggesting that MIP-1β secretion by HCV monocytes participates in the Th1 defect of DC.

**Conclusions:**

Our data indicate that monocytes from HCV patients are activated in vivo. This interferes with their differentiation into DC, leading to deficient TLR4 signaling in these cells that are enable to induce a Th1 response. This specific defect is linked to the activation of the MEK/ERK pathway.

## Introduction

Hepatitis C virus (HCV) is a single-stranded RNA virus classified in the Flaviviridae virus family by sequence homology [1], [2]. HCV infection is a major public health problem since 200 millions people are infected worldwide. The infection is characterized by a high rate of progression to chronicity which often leads to hepatic diseases such as cirrhosis and hepatocellular carcinoma [3]. In chronically infected patients, HCV often resists to clearance even after repeated antiviral treatments. Thus, the virus may have evolved strategies to escape the immune surveillance.

Several groups have reported that monocyte-derived DC (MoDC) from HCV chronically-infected patients have impaired allostimulatory capacities [4]–[11], however this was challenged by other groups showing that MoDC from chronically-infected patients could phenotypically and functionally mature [12]–[14]. Since the patients appear immunologically competent, major dysfunction of dendritic cells (DC) is not expected, rather a discrete DC function could be targeted by the virus without affecting global immunity. Recent studies reported that peripheral blood DC of chronically infected patients express less IL-12 than control cells from healthy individuals [15] and a small population of circulating myeloid DC displayed an impaired response to specific toll-like receptor (TLR) stimulation [16]. These transmembrane receptors expressed by cells of innate immunity are involved in the detection of microbes and activation of signaling pathways inducing the innate immune response.

There is increasing evidence that viruses can interact with TLR signaling. Viral components can either bind to TLR and activate their signaling pathway or block TLR function by interfering with intracellular intermediates. TLR3 activation can be triggered by polyriboinosinic-polyribocytidylic acid (poly I:C), a synthetic analog of double-stranded RNA (dsRNA) generated during viral replication. Moreover, the HCV serine protease NS3/4A can specifically degrade the adaptor protein TRIF resulting in inhibition of TLR3 signaling in HeLa cells [17]. TLR2 is also involved in the detection of viral components such as measles hemagglutinin or viruses such as human cytomegalovirus or herpes simplex virus type 1 [18]–[20]. Although TLR4 is well-known for its capacity to detect bacterial lipopolysaccharide (LPS), this receptor can also recognize viral components such as the fusion protein from respiratory syncytial virus (RSV) [21] and the envelope protein of mouse mammary tumor virus (MMTV) [22]. TLR4 is important for clearing RSV infection [23] whereas MMTV maintenance depends on TLR4 stimulation by the virus that induces immunoregulatory IL-10 secretion [24]. HCV non structural proteins such as NS5A can interact with MyD88, inhibiting TLR signaling and reducing cytokine production resulting from TLR activation in macrophages [25].

We examined the type of T cell response elicited by myeloid DC derived from blood monocytes after stimulation with different TLR ligands. Here we show that DC differentiated from monocytes of patients chronically infected by HCV present an altered TLR4-induced maturation resulting in a deficient Th1 function that can be restored by inhibition of the MEK-ERK signaling pathway. The monocytes isolated from these patients have an increased level of activation and secrete higher amounts of cytokines and chemokines especially MIP-1 α and β, that can interfere with DC functional maturation.

## Materials and Methods

### Patients

Blood samples were obtained from volunteers attending the Liver Unit at Necker Hospital (Paris, France). Blood was collected with the written informed consent of the patient during a therapeutical bleeding prescribed by Pr. S. Pol (Service d'hepatologie, Hôpital Cochin, Paris). Clinical protocols conformed to ethical guidelines of the authors' institutions. Chronically-infected patients have not been given antiviral therapy for more than six months. Viruses were of genotypes 1a, 1b, 3 and 4a.

### Monocyte purification and stimulation

CD14^+^ monocytes were purified from peripheral blood of healthy or chronically-infected individuals mainly as previously described [26]. PBMC were isolated from human peripheral blood by standard density gradient centrifugation on Ficoll-Hypaque (GE healthcare, Uppsala, Sweden), then mononuclear cells were separated from PBL by centrifugation on a 50 % Percoll solution (GE healthcare). Monocytes were purified by immunomagnetic depletion (Dynal, Oslo, Norway), using a cocktail of mAbs anti-CD19 (4G7 hybridoma), anti-CD3 (OKT3, American Type Culture Collection, Rockville, MD, USA), anti-CD56 (NKH1, Beckman Coulter, Fullerton, CA, USA) and anti-CD16 (Beckman Coulter). Recovered monocytes were more than 90 % pure as assessed by CD14 labeling. Monocytes (10^6^ cells/ml) could be stimulated for 24h in complete RPMI 1640 medium supplemented with 10% FCS, with either 1 µg/ml LPS (E. coli 055:B8, Sigma-Aldrich), 10 µg/ml poly I:C (pIC; GE healthcare), 10 µg/ml peptidoglycan (PGN; S. Aureus, Sigma-Aldrich) or 10 µg/ml Pam3CSK4 (Pam; Axxora, San Diego, CA). Cells were pelleted and supernatants harvested.

### HCV-RNA quantification

RNA was extracted from 10^6^ monocytes or PBL, using RNeasy mini kit (Qiagen, Courtaboeuf, France). RNA was eluted in 40 µl water and stored at -80°C. HCV-RNA quantification was performed by real-time PCR of the 5′ HCV non-coding region as described previously, but with minor modifications [27], [28]. Briefly, RNA (4 µl) was reverse-transcribed with a Thermoscript reverse transcriptase kit (Gibco-BRL) with the RC21 primer [29]. Real-time PCR was carried out with 2 µl cDNA and the RC1 and RC21 primers by using an LC FastStart DNA Master SYBR Green I kit and a LightCycler apparatus (Roche Diagnostics).

### DC differentiation and maturation

Purified monocytes (10^6^ cells/ml) were differentiated to immature DC in complete RPMI 1640 medium supplemented with 10% FCS, 50 ng/ml human rGM-CSF and 62.5 ng/ml human rIL-4 (from AbCys, Paris, France) during 6 days. Maturation was induced at day 5 with LPS (1 µg/ml), PGN (10 µg/ml), Pam (10 µg/ml) or pIC (10 µg/ml). When indicated, cells were pre-incubated with 40 µM MEK inhibitor (PD98059; Biomol, Plymouth Meeting, PA, USA) at day 4, or with 200 ng/ml MIP-1β or 20 ng/ml MIP-1α (R&D systems, Minneapolis, MN, USA) at day 0. All cells and culture supernatants were collected at day 6.

### Phenotype

Phenotype was analyzed by flow cytometry on a FACSCalibur (BD Biosciences, Franklin Lakes, NJ) using FITC-conjugated anti-CD14, -HLA-DR, -CD80, -CD54 and PE-conjugated anti-CD1a, -CD86, -CD83 and -CD40 (all from Beckman Coulter).

### Cytokine assay

Culture supernatants were stored at –80°C. Cytokine concentrations were determined using sandwich ELISA specific for IL12p40 (Biosource, Camarillo, CA, USA), IL-6, TNFα, IL-10 and IL-13 (Endogen, Woburn, MA, USA) or using Cytometric Bead Array (CBA) inflammation kit or CBA flex (BD biosciences) for IFNγ, IL-5, IP-10, MIP-1α, and MIP-1β.

### Mixed Lymphocyte Reaction (MLR)

Allogeneic CD3^+^ T cells were purified from non-infected blood donors as described [26] and primary MLR were conducted in 96-well flat-bottom culture plates. DC recovered at day 6 were washed and cocultured with 2×10^5^ allogeneic T cells at DC/T cell ratios ranging from 1/10 to 1/40. Culture supernatants were harvested after 5 d of coculture for IL-5, IL-13 and IFNγ assay.

## Results

### Deficient Th1 function in MoDC from HCV patients upon LPS stimulation

**Figure 1:**
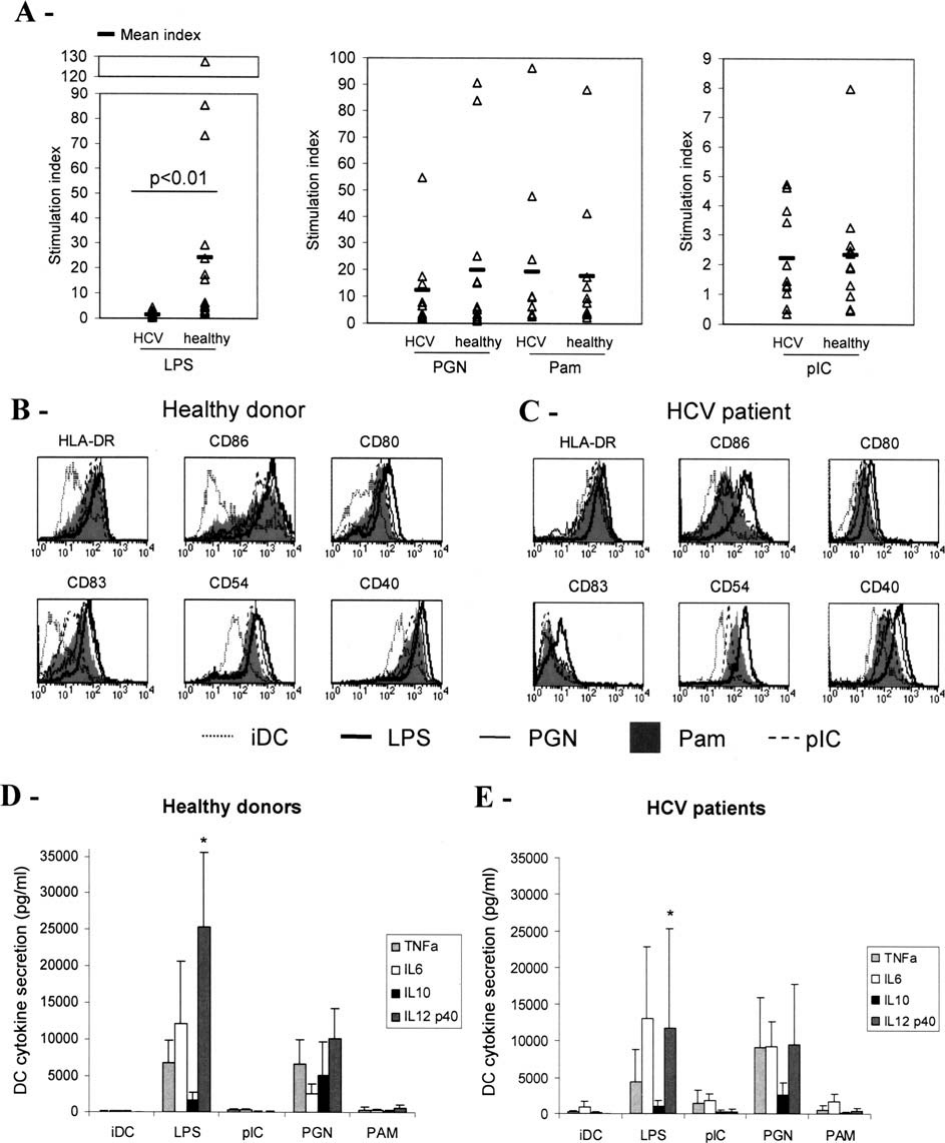
Comparison of MoDC from chronically HCV infected patients and healthy individuals upon TLR ligands stimulation. MoDC from healthy individuals or chronically HCV infected patients were treated at day 5 with LPS, PGN, Pam or pIC for 24h. Control immature DC were obtained at day 6 without any treatment. (A) DC harvested at day 6 were cocultured for 5 days with allogeneic T cells at a ratio of 1/10 DC/T cell in triplicates. IFNγ was assayed in coculture supernatants using CBA Flex kit. Stimulation index was determined as the ratio between stimulated DC and control iDC of the mean IFNγ secretion for the triplicate. The Mann-Whitney U test was used to generate p value for comparison of HCV (n = 11) versus healthy (m = 17) individuals. The mean stimulation index for both groups is indicated on the graph and median values for healthy donors and HCV patients were respectively 1.1 and 6.2 with LPS-stimulated DC, 7.2 and 6.0 with PGN-stimulated DC, 8.1 and 7.9 with PAM-stimulated DC and 1.4 and 1.9 with pIC-stimulated DC. (B, C) Phenotypic analysis were performed on DC harvested at day 6 from an healthy donor (B) or an HCV patient (C). Representative results of one experiment out of eleven. (D, E) Cytokine secretions in DC supernatants harvested at day 6. Mean secretion±SD for eleven different HCV patients and 10 to 18 healthy donors. * The mean secretion of Mo-DC from HCV patients is lower than that of MoDC from healthy individuals with p<0.01 in a Student's test.

The capacity of MoDC from chronically-infected patients to stimulate allogeneic T cells was examined by MLR experiments and compared to MoDC from healthy blood donors. Allogeneic T cells from healthy donors were used to assess MoDC ability to induce a Th1 or Th2 oriented response, upon activation by different TLR ligands. LPS was used as a prototype of TLR4 ligand, pIC for TLR3 stimulation, and PGN or Pam as ligands of the heterodimers TLR2/TLR6 or TLR2/TLR1 respectively. MoDC from chronically-infected patients stimulated with the TLR4 ligand LPS showed a strongly decreased ability to induce IFNγ secretion by allogeneic T cells compared to MoDC from healthy individuals (figure 1A). These LPS-treated MoDC were only slightly more efficient than immature MoDC with a mean stimulation index of 1.6 compared to 24.3 for MoDC from healthy donors. This difference between MoDC from HCV and healthy individuals was statistically highly significant (p<0.01) using the non parametric Mann-Whitney rank test. No significant increase in Th2 cytokines such as IL-5 and IL-13 could be detected in coculture supernatants of T cells with HCV-MoDC compared with healthy MoDC (data not shown). Maturation of MoDC from HCV-infected patients stimulated by TLR2/6, TLR2/1 or TLR3 ligands yielded cells able to stimulate IFNγ production by T cells as efficiently as MoDC from healthy individuals (figure 1A). Therefore HCV infection is associated with a selective Th1 defect of MoDC in the TLR4 pathway. To understand how HCV infection interferes with TLR4-induced maturation, we analyzed the phenotype and the cytokine secretion of mature cells.

The expression of characteristic markers of DC maturation was upregulated on MoDC in response to TLR ligand stimulation (figure 1B, C). Without any stimulation, MoDC from HCV chronically-infected patients had the characteristics of immature DC. In response to TLR ligands, DC maturation resulted in enhanced expression of costimulation molecules (CD80, CD86, CD40), MHC class II and adhesion molecules (CD54) with similar efficiencies whether cells were derived from HCV patients or healthy donors. Differences in HLA-DR and CD86 expression levels between healthy and HCV^+^ individuals are donor-dependent (figure S1). Therefore the deficient Th1 function of LPS stimulated MoDC from HCV patients could not be explained by altered expression of these surface markers of DC.

**Figure 2:**
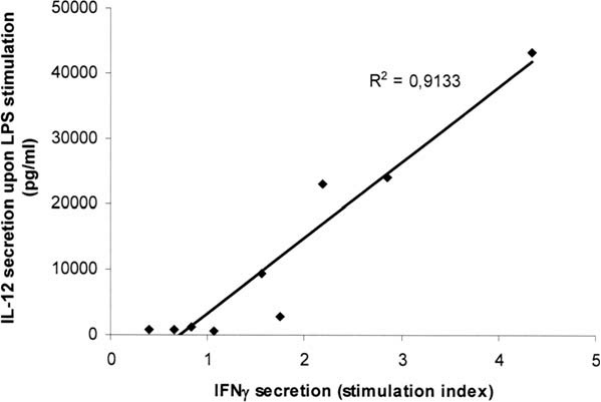
Decreased IL-12 secretion by MoDC from HCV-chronically infected patients is correlated to Th1 deficient function. MoDC from HCV chronically infected patients were treated at day 5 with LPS and harvested at day 6. IL-12p40 was quantified in the supernatants and DC were cocultured for 5 days with allogeneic T cells at a ratio of 1/10 DC/T cell. IFNγ was measured by CBA in supernatants of cocultures. Stimulation index was determined as the ratio of mean triplicate value of IFNγ induced by LPS stimulated DC compared to control iDC. R^2^ is Pearson's correlation coefficient.

The cytokine secretion profile of mature DC participates in the orientation of the T cell response. Therefore we quantified the cytokines secreted in the supernatant of DC culture after 24 h maturation induced by the different TLR ligands. Pam or pIC-stimulated DC secreted low amounts of IL-6, TNFα, IL-10 and IL-12 whereas LPS or PGN-activated DC showed strong secretions of cytokines especially IL-6 and IL-12 (figure 1D, E). A significant difference could be observed for IL-12 secretion. Upon LPS stimulation, MoDC from chronically-infected individuals secreted lower amounts of IL-12 than MoDC from healthy individuals (p<0.01). Thus Th1 disabled function following TLR4 stimulation appeared to be correlated to low IL-12 secretion by DC derived from HCV patients (figure 2). IFNγ secretion induced by LPS-stimulated MoDC from healthy donors was not correlated to the amount of secreted IL-12, indicating that in normal MoDC, IL-12 is not a limiting factor of the induction of the Th1 response.

### Th1 defect is not correlated to HCV-RNA content in monocytes

**Figure 3:**
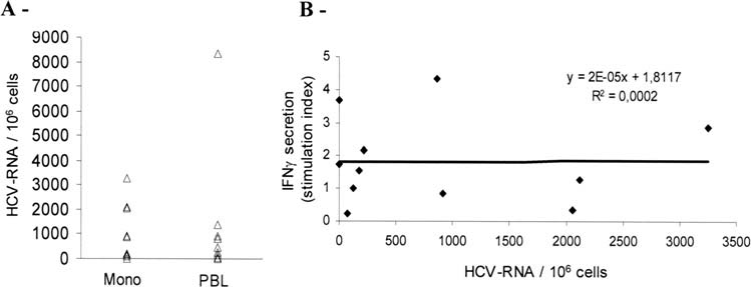
HCV-RNA content in monocytes does not correlate with Th1 defect of MoDC from HCV patients. (A) Quantification by RT-PCR of the positive strand of HCV-RNA extracted from 10^6^ monocytes or PBL from 11 distinct HCV chronically infected patients. RNA extraction was realized in triplicate and the mean value of three quantifications is shown. (B) HCV-RNA content in monocytes was plotted against the stimulation index obtained for each patient. Stimulation index was calculated as the ratio of IFNγ secretion by T cells induced by LPS-treated DC compared to iDC in allogeneic MLR experiments realized as described in figure 1A. HCV-RNA in monocytes and the stimulation index are not linearly correlated with a Pearson correlation coefficient R^2^ close to zero.

The presence of HCV-RNA in monocytes of patients has been previously described and was confirmed by our results. However, we found a very low level of HCV-RNA in these cells with less than 4 copies of HCV-RNA for 10^3^ monocytes (figure 3A). HCV-RNA content in PBL was even lower. Moreover we did not observe any correlation of HCV-RNA content in blood monocytes with the intensity of the Th1 defect upon LPS stimulation (figure 3B). After differentiation of monocytes into DC, HCV-RNA level was extremely low and at the limit of the detection threshold (data not shown). Therefore, it is very unlikely that the presence of HCV-RNA in monocytes or in DC could be responsible for the Th1 defect.

### Th1 function can be restored by inhibition of the MEK-ERK pathway

**Figure 4:**
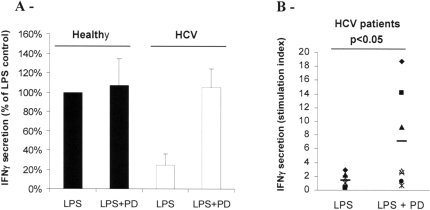
ERK inhibition can restore the Th1 function of MoDC from chronically-infected HCV patients stimulated by LPS. MoDC from healthy individuals or HCV chronically infected patients were treated at day 5 with LPS, 24h after addition of the ERK pathway inhibitor PD. DC harvested at day 6 were cocultured for 5 days with allogeneic T cells at a ratio of 1/10 DC/T cell. IFNγ was measured by CBA in supernatants of cocultures. (A) Comparison of MoDC from one healthy donor (filled bars) and one HCV patient (opened bars) in a MLR using the same allogeneic T cells. IFNγ secretion was normalized to 100% for control healthy LPS-treated MoDC. Mean±SD of three independent experiments in triplicates. (B) Treatment with the ERK inhibitor PD of MoDC from 7 chronically-infected HCV patients before LPS stimulation. Stimulation index was determined as the ratio of mean triplicate value of IFNγ induced by LPS-stimulated DC compared to control iDC. Statistical analysis was performed using the Student's test for paired data.

Since DC differentiation occur in absence of viral components, monocytes may have been conditioned in HCV patients by signals affecting their differentiation into mature DC. p38-MAPK and ERK kinases have been shown to play a role in TLR4 maturation of DC [30]–[34]. We previously found that the hyperactivation of ERK pathway could disable the Th1 function of DC treated by 1-methyl-tryptophan [35]. Therefore the MEK inhibitor PD98059 (PD) that prevents ERK phosphorylation was used to analyze the involvement of this pathway in the Th1 defect of HCV MoDC. These cells were treated at day 4 with PD before LPS stimulation at day 5. DC harvested at day 6 were washed and co-cultured with allogeneic T cells and IFNγ secretion was quantified. The results show that blocking the MEK-ERK pathway in MoDC from HCV chronically-infected patients restored their ability to induce IFNγ secretion by T cells (figure 4A). The restoration has been observed for 6 out of 7 patients. For patients DC that responded to ERK inhibitor treatment, the stimulation index for IFNγ secretion was increased by more than 5 fold in mean (figure 4B). These results suggest that monocytes from HCV patients display an altered ERK pathway that can interfere with LPS-induced maturation.

### Monocytes from HCV patients secrete higher amounts of cytokines and chemokines

**Figure 5:**
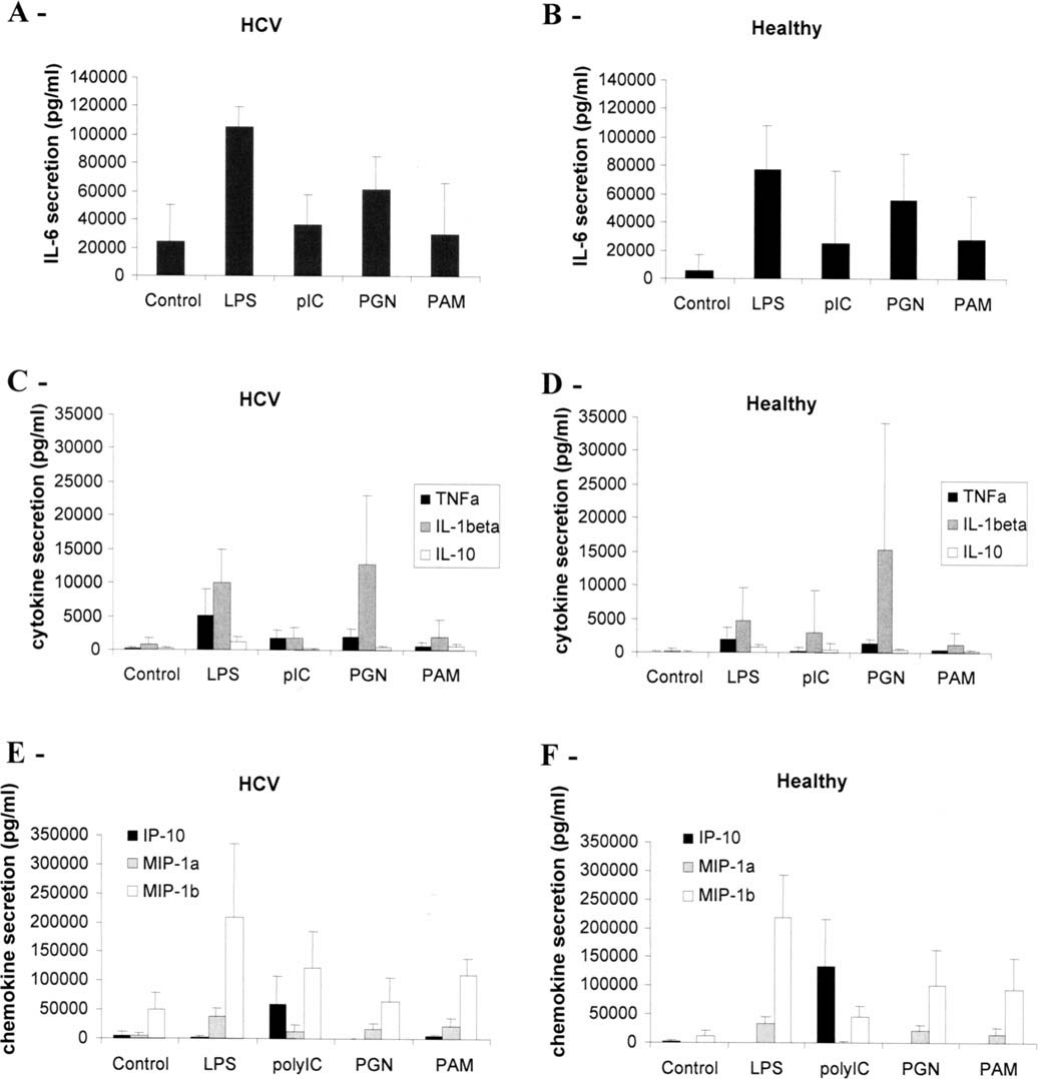
Cytokine and chemokine secretions of monocytes. Monocytes were isolated from HCV chronically-infected patients (A, C, E) or healthy individuals (B, D, F). They were incubated for 24h in the presence or not (control) of TLR ligands. Cytokines (A–D) and chemokines (E, F) were measured in the supernatants using CBA inflammation kit for IL-6, TNFα, IL-1β and IL-10 and specific CBA Flex kits for IP-10, MIP-1α and MIP-1β. Mean secretion±SD for five different HCV patients and healthy donors is shown.

**Figure 6:**
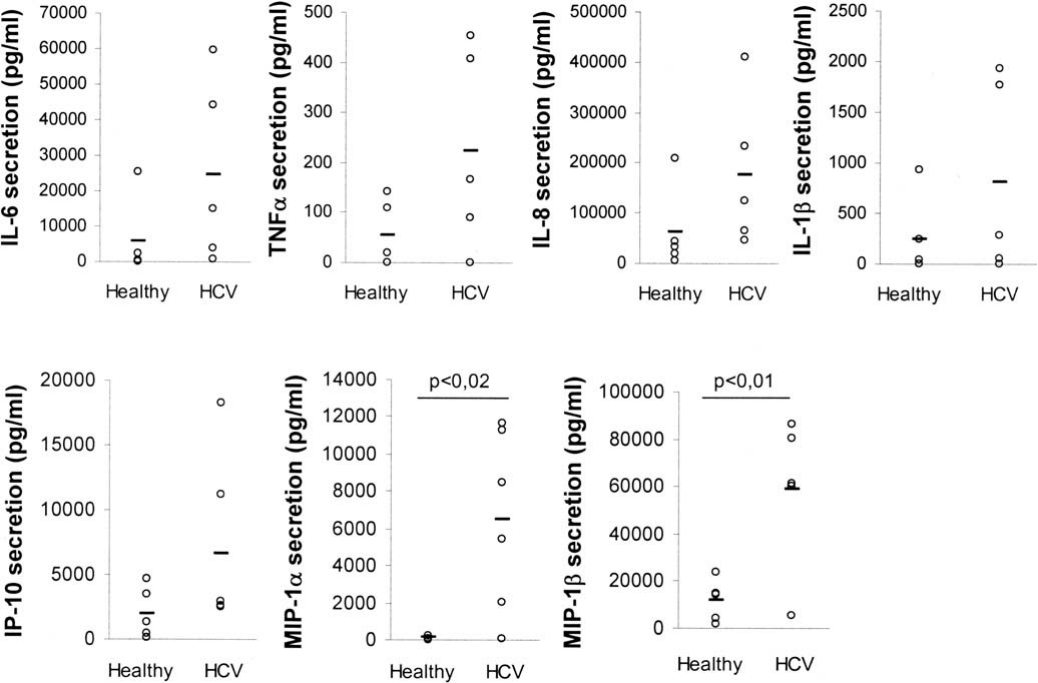
Cytokine or chemokine secretion by non-stimulated monocytes. Monocytes were isolated from HCV chronically-infected patients or healthy individuals. They were incubated for 24h in culture medium. Cytokines and chemokines were measured in the supernatants using CBA inflammation kit and specific CBA Flex kits respectively. Individual secretion values for five different HCV patients and healthy donors are shown by circles and the mean secretion is marked by the line. Statistical analysis was performed using the Student's test.

To analyze the activation state of monocytes, we measured their capacity to secrete cytokines and chemokines without stimulation or in response to a 24h-treatment with TLR ligands. We did not found any significant difference in the response to TLR ligands of monocytes from HCV patients compared to those isolated from healthy individuals (figure 5) as assessed by their similar levels of secretion of IL-6, IL-1β, TNFα, IL-10, IP-10 and MIP-1α and β. Especially no defect in the response of HCV-monocytes to TLR4 ligand could be detected. In contrast a difference was observed on non-stimulated monocytes as their basal level of cytokine and chemokine secretion was higher for HCV patients than for healthy individuals (figure 6). The difference is especially important for MIP-1 α and β, IL-6 and IL-8. The global increase in all cytokines and chemokines measured, strongly suggests that monocytes from HCV patients have an elevated activation state probably resulting from environmental modifications induced by HCV infection. This increased level of cytokine secretion may interfere with DC differentiation resulting in a Th1 defect in their response to LPS.

### MIP-1β interferes with monocyte differentiation into DC, leading to Th1 disabled DC

**Figure 7:**
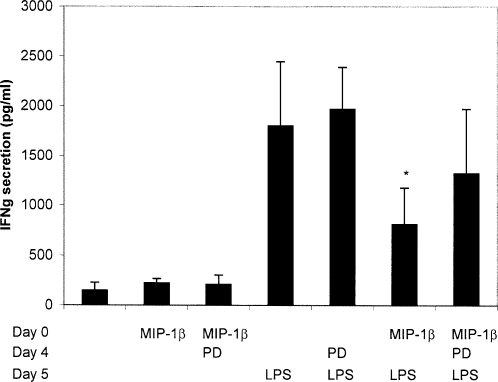
MIP-1β interfere with monocyte differentiation into DC. Monocytes isolated from an healthy individual were differentiated into DC in presence or not of 200 ng/ml MIP-1β. When indicated, monocytes were treated with the MEK inhibitor (PD) at day 4 and maturation was induced by LPS at day 5. Cells harvested at day 6, were cocultured for 5 days with allogeneic T cells at a ratio of 1/10 DC/T cell in triplicates. IFNγ was measured by CBA in supernatant of cocultures. Mean IFNγ secretion±SD of triplicate wells is shown. * The mean IFNγ secretion stimulated by Mo-DC treated with MIP-1β and LPS is significantly reduced compared to that of MoDC treated with LPS with p<0.05 in a Student's test.

MIP-1α and β can both stimulate CCR5, resulting in ERK activation. We analyzed the impact of MIP-1 β that can be secreted by monocytes on DC differentiation. Monocytes from healthy individuals were differentiated in DC with GM-CSF and IL-4 in the presence of 200 ng/ml MIP-1β. DC collected at day 6, after 24 h maturation with LPS, presented a reduced ability to induce IFNγ secretion by T cells compared to DC differentiated without MIP-1β (figure 7). This Th1 deficient function of DC could be partially restored by pre-treatment with the MEK inhibitor 24 h prior to LPS maturation. A similar tendency was observed for MIP-1α although data were not statistically significative (data not shown). These results show that introduction of MIP-1 at early stage of differentiation of MoDC results in DC with reduced Th1 function in response to LPS stimulation, a mechanism involving the MEK/ERK pathway.

## Discussion

In this paper, we observed that MoDC from HCV chronically infected patients presented a defective ability to stimulate IFNγ secretion by T cells upon LPS maturation. The allostimulatory defect was observed only when DC were stimulated with the TLR4 ligand LPS and not with TLR3, TLR2/6 or TLR1/2 ligands. A growing list of molecules from bacteria, viruses, and parasites can interfere with TLR4 signaling. This underlines the crucial role of TLR4 pathway in the innate recognition of pathogens but may also reveal microbial strategies evolved to facilitate infection. Physiological relevance of TLR4 signaling interference by HCV may relate to liver and intestine infection [36], [37]. The liver lies directly downstream of the gut and is therefore constantly exposed to antigens and microbial products derived from the intestine. The control of liver inflammation and tolerance to gut-derived antigens (food antigens and commensal bacteria) is likely to rely, at least in part, on functional characteristics of hepatic and intestinal antigen presenting cells. One of these characteristics is the modulation of TLR4 signaling under the dual pressure of protecting the host from pathogenic infections and coexistence with the myriad commensal organisms [38].

There are several reasons for HCV to interfere preferentially with the TLR4 pathway. This could first reflect the action of LVP on monocytes. HCV can circulate in the blood of chronically infected patients in the form of Lipo-Viro-Particles (LVP) [27], [37], [39]–[41]. These particles are triglyceride-rich lipoproteins, containing viral RNA and capsids and carrying the viral envelope proteins at their surface. LVP are found in all chronically infected patients and can represent a variable proportion of the total viral load ranging from a few percent to 90 percent. They originate from both the liver and the intestine [37]. We previously reported that LVP can interfere with TLR4-triggered maturation of DC, resulting in cells having a better potential to stimulate a Th2 response rather than a Th1 response of T cells. This shift of DC function did not occur upon pIC stimulation and was found to be dependent on ERK and p-38-MAPK activation [34]. Although MoDC from HCV patients did not acquire a Th2 function, one intriguing possibility is that LVP could interact with blood monocytes and modify their capacities to differentiate into DC. The impact of LVP on blood monocytes remains to be analyzed but it could be mediated either by the viral material carried by the particle or by its lipid contain. It was previously shown that HCV E2 envelope protein can induce ERK and p-38-MAPK signaling in human T cells via both CD81 and Low-Density Lipoprotein Receptor [42]. LVP could also contain modified lipids that could activate ERK or alter lipid raft distribution, thus inhibiting subsequent LPS maturation of DC ex vivo [43]–[46]. It has been shown recently that lipoproteins from HCV patients induced some modifications of lipid synthesis in human monocyte-derived macrophages [47]. LVP could thus interfere with TLR4 signaling by modifying the lipid composition of the plasma membrane of monocytes. Among other possibilities, the induction of Th1 responses can be inhibited by polyunsaturated fatty acids (PUFA), possibly by insertion in the membrane [48]. PUFA are known to be agonists of PPARγ and we have previously shown that activating PPARγ in DC can inhibit their functional maturation, leading to phenotypically mature DC unable to induce a Th1 response of T cells [49].

In MoDC from HCV patients, TLR4 signaling remains functional since the ability of DC to induce IFNγ secretion by T cells can be restored by treatment with the MEK inhibitor 24 h prior to LPS maturation. The RAS/MEK/ERK pathway is involved in the regulation of differentiation in many cell types. However, the function of this pathway depends on the cell type, the differentiation stage of the cell and the type of stimulation of the receptor. Interestingly, ERK phosphorylation is involved in the inhibition of the B cell response to TLR4 agonist LPS [50]. We previously observed that an increased phosphorylation of p38 and ERK MAP-kinases and a sustained activation of the transcription factor c-Fos were correlated to a Th2 shift of DC function [35]. In this model of functional shift induced by 1-methyl-tryptophan, we found that ERK inhibition could restore the ability of DC to stimulate IFNγ secretion by T cells. This was also the case for DC generated from HCV patients as shown here. This result strongly suggests that monocytes isolated from the blood of these patients present some modifications resulting in a deregulation of ERK phosphorylation. This dysfunction can be due either to a positive stimulation of the MEK/ERK pathway or to the absence of negative regulation by phosphatases such as the dual-specific phosphatases (DUSP) that oppose the action of MEK by dephosphorylating ERK. This latter mechanism has been observed for TLR4-induced plasma cell differentiation [50].

The deficient response to LPS stimulation of MoDC points at an alteration of the monocytes isolated from HCV patients. This defect is not correlated to the amount of HCV-RNA detected in monocytes. The low content of viral RNA in these cells (less than 4 copies for 10^3^ cells) is in favor of a role of environmental factors affecting monocytes function. No or trace amounts of HCV-RNA remained in differentiated DC indicating that they probably did not present HCV peptides. In consequence, these cells should not specifically display an HCV-specific defect in term of antigen presentation and activation of HCV-specific T cells. We also found that the addition of IFNα 24h prior induction of maturation by LPS slightly improved the ability of DC differentiated from monocytes of 4 out of 6 HCV infected patients, to stimulate IFNγ secretion by T cells (data not shown). This weak restoration of the TLR4 stimulation efficiency is thus independent of the antiviral effect of IFNα and probably relate to signaling events triggered by IFNα.

Monocytes from HCV patients can respond to LPS stimulation, secreting cytokines and chemokines with an efficiency similar to that of control monocytes. Desensitization of these cells to LPS can therefore be excluded. However, we observed that monocytes from HCV patients had an elevated basal activation state compared to monocytes from healthy donors. HCV-monocytes secreted higher levels of cytokines and chemokines among which, MIP-1 α and β. DC differentiated from healthy monocytes in the presence of MIP-1β, displayed a similar Th1 defect after LPS stimulation than MoDC from HCV patients. This defect could be overcome by inhibition of the ERK pathway. Addition of anti-MIP-1 antibodies during differentiation of MoDC from HCV patients tend to restore the ability of these cells to stimulate a Th1 response (Figure S2). The MIP-1 α and β chemokines are found in higher amounts in the blood of HCV patients [51] and can both stimulate the CCR5 receptor. Although the mechanism involving the ERK pathway remains to be determined, ERK hyperactivation could result from CCR5 stimulation by MIP-1 as it was previously described for human macrophages [52]. Increased MIP-1 secretion by HCV monocytes may thus interfere with DC differentiation in vivo.

Several recent papers also described Th1 deficient functions of circulating myeloid DC in specific conditions depending on the activation pathway of DC [8], [16], [53]. Recently, it has been shown that the core protein of HCV can inhibit the Th1 function of monocytes-derived DC via interaction with gC1q receptor [54]. LPS-stimulated MoDC from HCV patients showed a mature phenotype but a significant decrease of IL-12 secretion that could participate to the Th1 defect since the intensity of the Th1 defect was correlated to the intensity of IL-12 decrease. This selective defect of MoDC derived from chronically-infected HCV patients is in agreement with the absence of immunodeficiency and is in line with the study by Rodrigue-Gervais et al. showing deficient response to LPS of circulating DC ex-vivo [16]. A deficient secretion of IL-12 was also reported for circulating DC from HCV patients ex-vivo [6], [16].

By acting on TLR4 pathway, HCV may thus exploit a natural protective mechanism of the liver and the intestine normally used to control inflammation and immunity to commensal microorganisms.

## Supporting Information

Figure S1 (2.54 MB TIF)

Figure S2 (2.18 MB TIF)
