# Supplementary figures and images for: Th1 Disabled Function in Response to TLR4 Stimulation of Monocyte-Derived DC from Patients Chronically-Infected by Hepatitis C Virus
Source: PLoS One. 2008 May 28;3(5):e2260. doi: 10.1371/journal.pone.0002260 (PMC2377338; doi:10.1371/journal.pone.0002260)

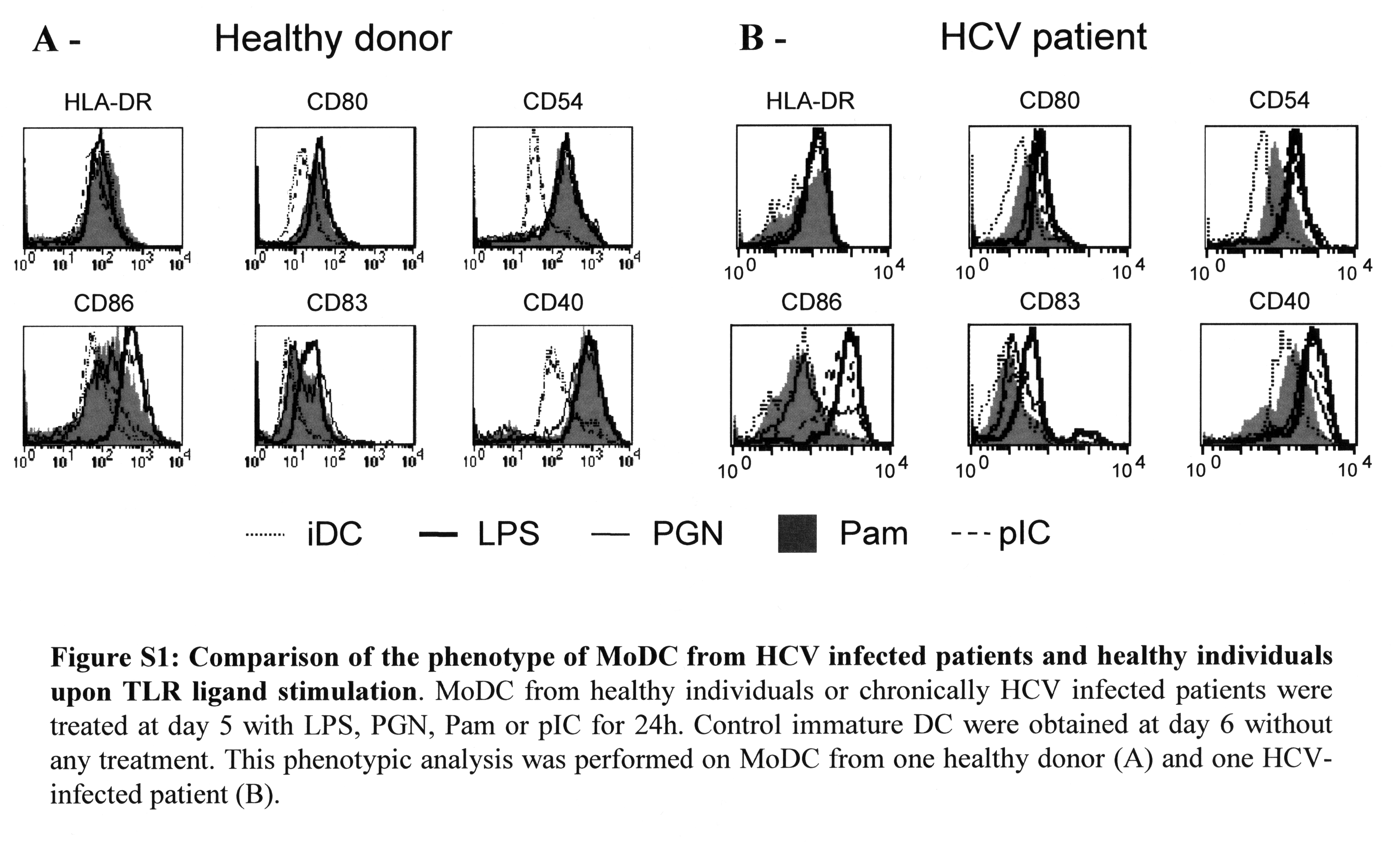

Supplement: Figure S1 — (2.54 MB TIF) [file pone.0002260.s001.tif]

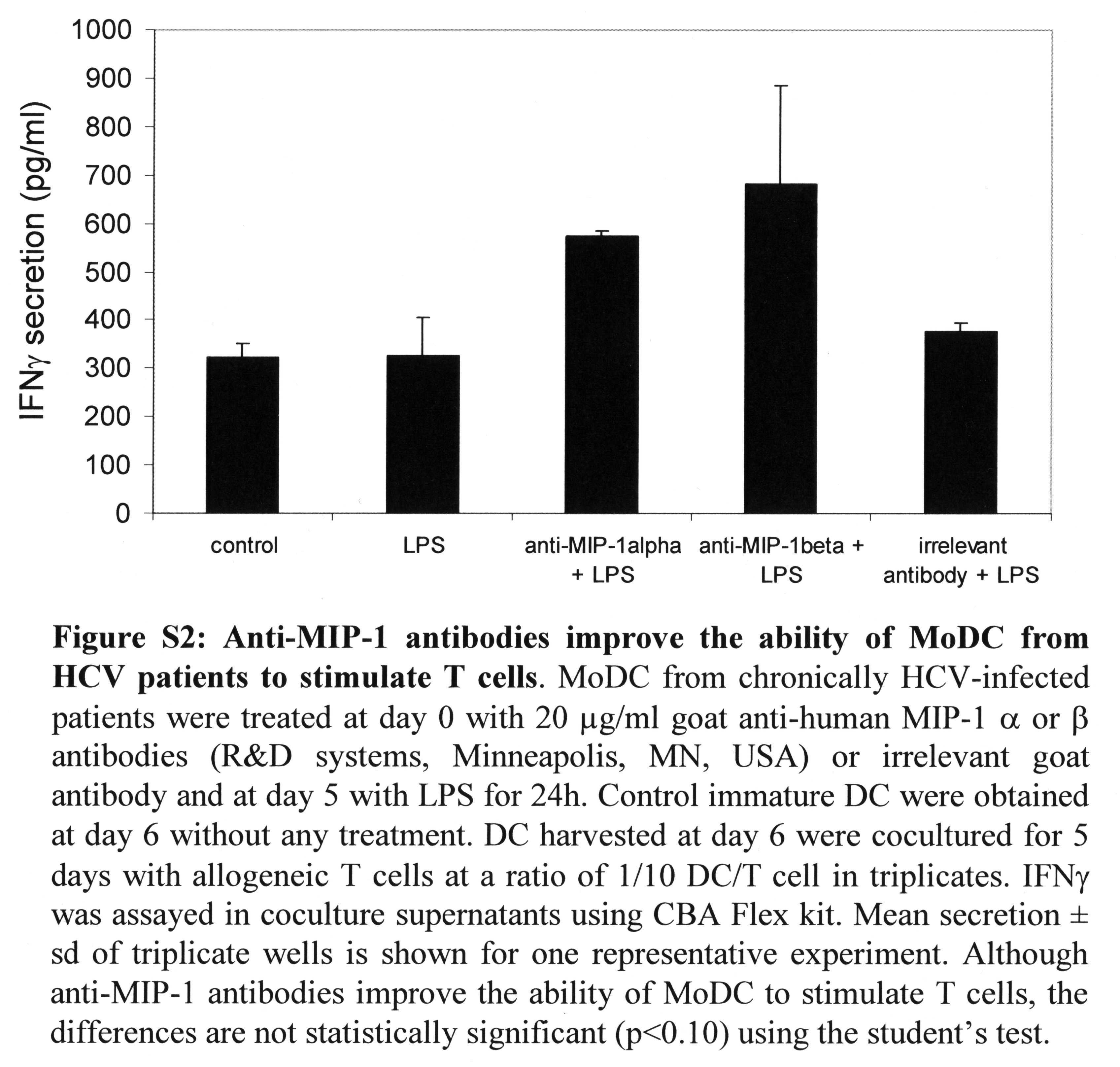

Supplement: Figure S2 — (2.18 MB TIF) [file pone.0002260.s002.tif]
